# The Outcome after Endovascular and Open Repair of Abdominal Aortic Aneurysms—A Binational Study Conducted between 1998 and 2017

**DOI:** 10.3390/jcm13154449

**Published:** 2024-07-29

**Authors:** Riku Pirinen, Matti T. Laine, Kevin Mani, Kim Gunnarsson, Anders Wanhainen, Reijo Sund, Maarit Venermo

**Affiliations:** 1 Department of Vascular Surgery, University of Helsinki, Helsinki University Hospital, 00290 Helsinki, Finland; matti.laine@hus.fi (M.T.L.); maarit.venermo@hus.fi (M.V.); 2 Department of Surgical Sciences, Uppsala University, 75185 Uppsala, Sweden; kevin.mani@uu.se (K.M.); kim.gunnarsson@regiongavleborg.se (K.G.); anders.wanhainen@uu.se (A.W.); 3 Kuopio Musculoskeletal Research Unit, Institute of Clinical Medicine, Surgery, University of Eastern Finland, 70211 Kuopio, Finland; reijo.sund@uef.fi

**Keywords:** aortic aneurysm, abdominal, endovascular repair, mortality, population, vascular surgery

## Abstract

**Objective**: We aimed to analyse patient outcomes following open (OAR) or endovascular repair (EVAR) of an abdominal aortic aneurysm (AAA) in Finland and Sweden from 1998 to 2017. Both intact and ruptured AAAs (rAAAs) were included in the analysis. **Methods**: Patient-level data from national registries in Finland and Sweden were analysed, pairing operations for intact and ruptured AAA repair with mortality data (date of death). All-cause mortality was the primary endpoint. Anonymized patient data from both countries were pooled, comprising a total of 32,324 operations. Ruptured and intact AAAs were considered separately. In total, EVAR was performed on 9619 intact AAAs and 1470 rAAAs, while OAR was performed on 13,241 intact AAAs and 7994 rAAAs. The patient’s age, sex and the date of operation were obtained as demographic information. Cox regression and Kaplan–Meier analyses were used to evaluate long-term (10-year) survival after the treatment of AAA or rAAA with either modality. Kaplan–Meier analysis was performed in three different age groups (<65 years, 65–79 years and ≥80 years). **Results**: Considering all age groups together, the 1-, 3- and 10-year Kaplan–Meier survival rates after EVAR were 93.4%, 80.5% and 35.3%, respectively, for intact AAA repair and 67.2%, 55.9% and 22.2%, respectively, for rAAA repair. For OAR of intact AAAs, the 1-, 3- and 10-year Kaplan–Meier survival rates were 92.1%, 84.8% and 48.7%, respectively. The respective rates for OAR of rAAAs were 55.4%, 49.3% and 24.6%. In a Cox regression analysis, a more recent year of operation was associated with improved survival, and older age affected survival negatively for both intact and ruptured AAA repair. If patients survived the first 90 days after the operation, the survival after intact AAA repair was 13.5 years for those <65 years (general population: 18.0 years), and 7.3 years for those ≥80 years (general population: 7.9 years). After rAAA repair, the mean survival was 13.1 years for patients <65 years and 5.5 years for patients ≥80 years, respectively. **Conclusions**: The long-term survival of patients undergoing intact AAA treatment at the age of 80 or older is close to that of the general population, provided they survive the operation. Conversely, for patients younger than 65, the long-term survival is markedly worse. The long-term survival of AAA patients has improved over time. Open surgery is still a safe and effective option for young patients undergoing intact AAA repair. Our results support the ESVS guidelines recommendation of EVAR being the first-line treatment for patients with rAAA.

## 1. Introduction

The following two surgical modalities exist for the treatment of an abdominal aortic aneurysm (AAA): open surgery (OAR) and endovascular repair (EVAR). EVAR was introduced in the 1990s and gained popularity due to its significantly lower invasiveness [1,2,3,4]. In Finland, the proportion of EVAR has increased steadily during the last decade [5]. EVAR has made AAA treatment accessible to patients who are ineligible for open surgery due to the risks associated with major surgical procedures. Indeed, the perioperative mortality associated with EVAR has been found to be lower than that of OAR in numerous randomized controlled trials [2,6,7,8,9]. In the follow-up studies of these same trials, EVAR has not been as durable as OAR in the long term, carrying a higher risk of rupture and reintervention [10].

In AAA repair, a high case volume has been associated with favourable outcomes in various healthcare systems [11,12,13]. While the aforementioned RCTs revolutionized AAA treatment, EVAR was a relatively uncommon operation at the start of these trials, and experienced high-volume centres did not exist. Furthermore, the trials used older-generation endografts with poorer performance than newer models [14].

In this study, we analysed patient mortality following OAR and EVAR in Finland and Sweden between 1998 and 2017, utilizing national registries from both countries. This binational approach was chosen to acquire a large dataset. Notably, these registries collectively span the entire populations of both nations, minimizing potential selection bias. The analysis included both intact and ruptured AAAs. As we did not have access to comorbidity or anatomical data (factors that are known to affect patient selection to EVAR or OAR), we do not aim to compare the treatment modalities, but to evaluate the long-term mortality after each treatment.

## 2. Methods

Patient-level data from Sweden and Finland were analysed from 1998 to 2017, allowing for 10 years of follow-up. All operations for the repair of intact and ruptured AAAs were identified from national registries and paired with mortality data from cause of death registries using personal identity codes. In Finland, the National Care Register for Health Care (HILMO) maintained by the Finnish Institute of Health and Welfare was used to identify all procedures performed to repair ruptured or intact AAAs, and the causes of death register by Statistics Finland was searched to identify dates of death. The HILMO register includes all inpatient and outpatient contacts, including all performed procedures, irrespective of the care provider, and the causes of death register contains data on all deaths in Finland. In Sweden, the National Patient Registry (NPR) was employed to identify all operations and the national cause of death registry for data on dates of death. The Swedish registries are maintained by the National Board of Health and Welfare (NBHW), and they contain data on all inpatient and outpatient care (NPR) and deaths, with the registration of diagnoses and surgical procedures based on standardized coding. The extraction of data was performed using diagnostic codes for rAAA and intact AAA (I71.3, I71.4), according to the International Classification of Diseases, 10th revision (ICD-10). Surgical procedures were assessed based on the NBHW Classification of Surgical Procedures and the Nordic Medico-Statistical Committee (NOMESCO) Classification of Surgical Procedures [15]. The NOMESCO codes used can be found in Table S1.

The data gathered included personal ID number, date of birth, date of death, sex, possible AAA diagnosis (ICD-10), possible rAAA diagnosis (ICD-10) and the operation(s) performed (NOMESCO codes) and their date. Cross-matching between the cause of death registries and their respective surgical procedure registries was conducted using personal ID numbers. These data were then anonymised.

Anonymised data from both countries were combined and analysed in accordance with the General Data Protection Regulation (GDPR) of the European Union. Only the first procedure of the study period was included in the analysis. Cox regression and Kaplan–Meier analysis were used for 10-year survival analysis. Follow-up started from the index procedure and ended at death (events) or on 31 December 2017 (censored) for all Finnish AAA and rAAA patients and for all Swedish rAAA patients. Swedish patients with an intact AAA who were operated on between 1998 and 2013 were followed until end of 2014, and those who were operated on from 2014 onwards were followed up until the end of 2017. For the Kaplan–Meier analysis, patients were divided into three age groups (<65 years, 65–79 years and ≥80 years) according to their age at the date of the primary operation.

SPSS 29.0 (IBM, Armonk, NY, USA) and STATA 16.0 (StataCorp LLC, Collage Station, TX, USA) were used for statistical analysis. Continuous variables are presented as mean and standard deviation and compared using Student’s *t*-test. The proportions are presented as percentages and compared using a Chi-square test. The mortality after intact AAA and ruptured AAA repair in the three different age groups is illustrated using Kaplan–Meier survival curves. Mortality data were analysed with the Cox regression model and adjusted by country, sex, age, treatment modality and year of primary operation. A *p* value <0.05 was considered significant.

The proportional hazard assumption was tested with a test based on Schoenfeld residuals that indicated potential non-proportionality, particularly for the endovascular vs. open repair variable in the intact AAA model (global test *p* < 0.001 for an intact AAA and *p* < 0.001 for a rAAA).

## 3. Results

A total of 22,860 intact AAA repairs and 9464 ruptured AAA repairs were included in the analysis. The proportions of women were 15.4% and 16.8%, respectively (*p* = 0.002). Patients with a ruptured aneurysm were slightly older than those with an intact aneurysm (72.3 vs. 74.0 years (*p* < 0.01); Table 1). The average duration of follow-up was 5.0 years (SD 4.3), with a median follow-up duration of 4.1 years (IQR 1.7–7.5).

The respective 1-, 3- and 10-year Kaplan–Meier survival rates after endovascular aneurysm repair were 93.4%, 80.5% and 35.3% for intact AAA repair and 67.2%, 55.9% and 22.2% for rAAA repair. Excluding 90-day mortality, the 1-, 3- and 10-year survival rates for AAA and rAAA patients treated with EVAR were 95.9% versus 91.7%; 82.7% versus 76.3%; and 36.3% versus 30.2%, respectively. The mean survival of the patients who were alive at the 90th postoperative day after EVAR for intact and ruptured aneurysm was 6.9 and 6.2 years, respectively (EVAR vs. rEVAR *p* < 0.001).

For open repair of intact AAAs, the 1-, 3- and 10-year Kaplan–Meier survival rates were 92.1%, 84.8% and 48.7%, respectively. The corresponding rates for OAR of rAAAs were 55.4%, 49.3% and 24.6%, respectively. Excluding 90-day mortality, the 1-, 3- and 10-year survival rates for AAA versus rAAA patients treated with OAR were 97.0% versus 94.5%; 89.3% versus 84.1%; and 51.3% versus 42.0%, respectively. The mean survival of the patients who were alive at the 90th postoperative day after OAR for intact and ruptured aneurysm was 7.8 and 7.1 years, respectively (OAR vs. rOAR *p* < 0.001).

**Figure 1:**
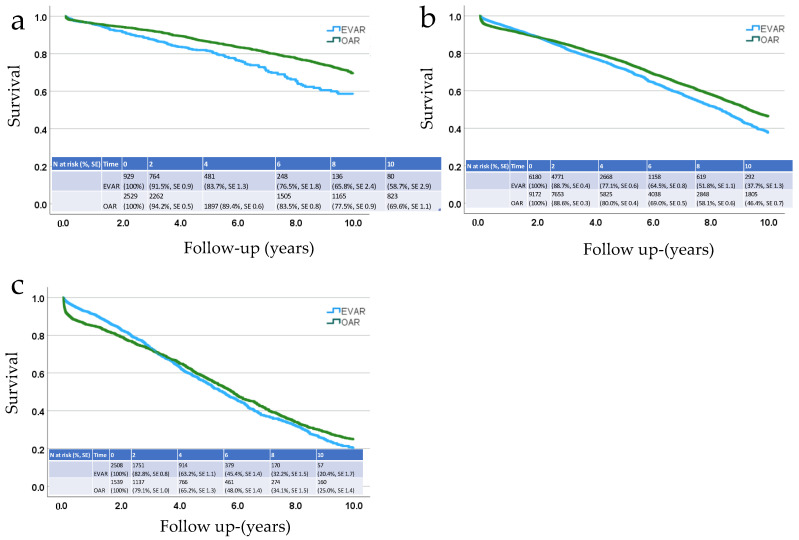
Kaplan–Meier analysis of 10-year survival after endovascular (EVAR) or open aneurysm repair (OAR) of an intact AAA in three age groups. (**a**) <65 years, (**b**) 65–79 years (**c**) ≥80 years. Survival percentages and standard errors (SEs) are presented after the numbers at risk.

**Figure 2:**
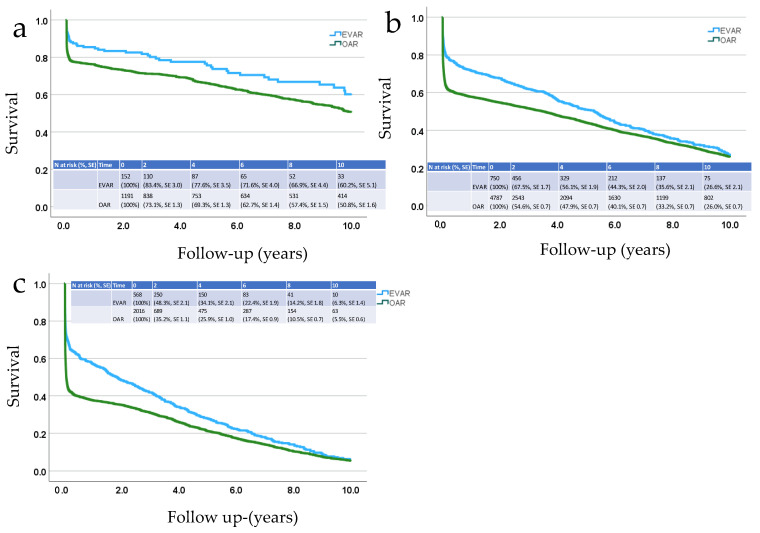
Kaplan–Meier analysis of long-term survival after endovascular (EVAR) or open aneurysm repair (OAR) of an rAAA in three age groups. (**a**) <65 years, (**b**) 65–79 years (**c**) ≥80 years. Survival percentages and standard errors (SEs) are presented after the numbers at risk.

The mean survival after EVAR for patients less than 65 years of age was 7.9 years (95% CI 7.6–8.2 years) after intact AAA repair and 7.5 (95% CI 6.9–8.1 years) after ruptured AAA repair; for patients aged 65–79 years, it was 7.9 (7.1–8.3 years) vs. 5.1 (5.4–5.3 years); and for patients >80 years old, it was 5.6 years (5.5–5.8 years) vs. 3.1 (2.7–3.4 years). The corresponding figures for OAR were 8.6 years (8.5–8.7 years) vs. 6.5 (6.3–6.8 years) for the youngest age group; 7.4 years (7.3–7.4 years) vs. 4.4 years (4.3–4.5 years) for the middle age group; and 5.7 (5.5–5.8 years) vs. 2.3 (2.1–2.4 years) for the oldest age group. The 10-year Kaplan–Meier survival curves in three different age groups (<65 years, 65–79 years and ≥80 years) are shown for intact AAA repair in Figure 1 and for rAAA repair in Figure 2.

In a Cox regression analysis, a more recent year of operation was associated with improved long-term survival, and older age affected survival negatively in the repair of both intact and ruptured AAAs. For intact AAAs, female sex and treatment with OAR were associated with improved long-term survival, but for rAAAs, the reverse was true (Table 2). Mean survival times for patients after intact AAA and rAAA repair are shown in Table 3.

## 4. Discussion

In the Cox regression, a later calendar year of the primary operation was associated with improved survival after both AAA and rAAA repair. Not surprisingly, age was the strongest predictor of long-term mortality after both AAA and rAAA treatment. Female sex and OAR were independently associated with better long-term survival after intact AAA repair but with poorer survival after rAAA repair. If patients survived the first 90 days after rAAA repair, their long-term survival was almost as good as that of patients after intact AAA repair, particularly in the youngest age group.

Considering the proportions of nationalities and sexes in our study population, the mean remaining life expectancy of the corresponding general population during the study period was 18.0 years at 65 years old, 12.1 years at 73 years old and 7.9 years at 80 years old [16,17]. Considering EVAR and OAR together, mean survival after intact AAA repair at the age of 80 years or older is close to the life expectancy of the general population of the same profile, if the operation is successful (i.e., if the patient survives the first 90 days after the operation). Conversely, in patients younger than 65 years of age, survival falls markedly short of the general population. This observation is probably explained by the comorbidities associated with an AAA, as well as smoking status, rather than the durability of the repair itself.

During the study period, frail patients with comorbidities were likely deemed unfit for OAR and treated with EVAR instead. Thus, the EVAR patients in this study are likely to have significantly more comorbidities than the OAR patients. On the other hand, patients with a rAAA who were selected for EVAR, particularly at the beginning of the study period, were likely hemodynamically stable at presentation and had a more favourable anatomy than patients selected for OAR. Because of this selection bias, no conclusions should be drawn on the superiority of either treatment option.

That being said, in the present study, EVAR yielded better survival for patients with rAAAs than OAR in all age groups during the follow-up period (Figure 2). This finding is consistent with the 3-year results from the IMPROVE trial [18]. Additionally, previous data suggest that the introduction of EVAR has resulted in lower turn-down rates for patients presenting to Swedish hospitals with an rAAA, thus contributing to a reduction in overall rAAA-related mortality within the population [19].

In the case of intact AAA repair, the Kaplan–Meier analysis shows better early survival for EVAR in patients aged 65 years and older (Figure 1). This difference disappeared after 2 to 3 years, and 10-year survival was poorer for patients treated with EVAR in all age groups except the over 80-year-olds. The advantage of EVAR in early survival is probably related to its low perioperative mortality in intact AAA repair. The disadvantage of EVAR in long-term survival in turn is related to the probable selection bias discussed earlier. Thus, EVAR patients are likely to have poorer overall survival than OAR patients regardless of AAA repair durability and EVAR should not be considered an inferior option for the treatment of an intact AAA due to these findings. Nevertheless, OAR does seem to provide a durable long-term solution in younger patients with an intact AAA.

Cox regression analysis also demonstrated that the country of treatment was a significant predictor of 10-year survival after intact AAA repair. This result was not observed for rAAA repair. For intact AAA repair, survival was better in Sweden than in Finland. Swedish patients were, perhaps, healthier than Finnish patients. During our study period, the average male life expectancy at 65 years was 0.95 years longer for the Swedish general population in comparison to Finland’s general population (17.80 years vs. 16.85 years) [19,20]. Furthermore, AAA screening was launched in Sweden during our study period in 2006. After screening, the mean aneurysm diameter in Sweden was probably smaller, which may positively impact mortality after the treatment of an intact AAA. Finally, the intact AAA caseload was greater in Sweden during our study period. Swedish AAA treatment centres may benefit from this high patient volume compared to their Finnish counterparts. Notably, Sweden also outnumbered Finland in rAAA cases, but no difference was observed in 10-year survival of rAAA patients between the countries.

Mao et al. published the results of intact AAA repairs from Australia, Germany and the United States from 2010 to 2017/18 [20]. The median age of their patients was 76–77 years, which is slightly older than the median age of 73 years of our patients treated during the same period. Mao et al. only included patients aged 65 years or older. In our patients, the median age after excluding those aged 65 or younger was 75 years. Use of EVAR was more common in the populations described by Mao et al. than in our study: 74.4–87.3% versus 61.6%, respectively. Their results on survival after EVAR versus open repair show similar trends to ours, with EVAR having an initial benefit and similar or poorer results in the long term, although they demonstrated some differences between the studied countries. The differences between EVAR and open repair, in terms of survival, were similar. The mortality rates from the three countries were similar to our results when we included only patients who were operated on from 2010 to 2017 and who were over 65 years of age. In our study, EVAR mortality at 60 days was 2.0% and OAR mortality was 4.4% compared to their reported mortality of 1.9–2.9% for EVAR and 5.5–8.4% for OAR. The respective 5-year mortality rates in our study were 32.1% and 25.6%, compared to their respective rates of 30.0–40.1% and 25.9–37.8%.

The strength of the current study is that it provides real-world data on AAA operations, capturing the entire population of two neighbouring countries using national registries. Patient-level data could be used for analysis, with reliable data on times of death. Additionally, the registers used in the study are mandatory for all healthcare providers in the two countries and capture very similar data, with personal ID numbers enabling precise cross-matching [21]. The populations of the two countries are genetically, culturally, and socio-economically similar, making it possible to analyse the data from both countries as one large cohort. Although there are differences in treatment traditions between the countries, these variations are comparable to those found between different centres within each country. Importantly, the study’s primary focus is not on comparing the countries, but on describing the outcomes of open surgical repair (OAR) and endovascular aneurysm repair (EVAR) in a large, combined population-based real-world dataset with a very high data quality.

The limitations of this study include the questions regarding the reliability of registry data [21]. The registries in question are nationwide, and the number of missed cases should therefore be minimal. Data in the registry can be mislabelled, but given the large number of cases in our study, the effect of mislabelling should be negligible when looking at large trends over a long period of time. Additionally, the proportional hazard assumption test based on Schoenfeld residuals indicated potential non-proportionality, particularly for the endovascular vs. open repair variable in the intact AAA model. This was expected and may lead to conservative bias (lower power to detect difference) in models assessing long-term survival. As discussed above, the limitation in comparing EVAR and OAR is that the available data do not allow us to adjust for comorbidities or aortic anatomy for the purposes of comparing the patient groups, and, thus, the patient groups cannot be adjusted according to the patients’ comorbidities. Another notable limitation of this study is that the study period ended in 2017. Since then, new EVAR devices have been introduced, and AAA treatment guidelines have evolved. The long-term outcomes for patients today are likely better than those during the study period. However, we believe this unique and large dataset effectively describes long-term trends in the survival of OAR and EVAR patients.

## 5. Conclusions

This study presents the long-term survival of intact AAA and rAAA patients treated with either EVAR or OAR in Finland and Sweden between 1998 and 2017. We used high-quality real-world data from large national registries of the two countries over a long period of follow-up. Our results show that long-term survival of patients after successful AAA treatment at the age of 80 years or older is close to that of the general population. Conversely, for patients younger than 65, the long-term survival is markedly worse. The long-term survival of AAA patients has improved over time. As we did not adjust for comorbidities, no conclusions should be drawn on the superiority of either treatment option. Nevertheless, our data suggest that open surgery continues to be a safe and effective option for young patients undergoing repair of an intact AAA. In the case of rAAAs, EVAR yielded good results in all age groups and, thus, our results support the recommendation of EVAR as the first-line treatment for rAAA outlined in current ESVS clinical guidelines [22].

## Figures and Tables

**Table 1:** Patient characteristics. Statistical significance between EVAR and OAR groups assessed by means of the Chi-squared test (% women) and the independent samples *t*-test (mean age).

	EVAR	OAR	All	
Intact AAA				
Number of operations	9619	13,241	22,860	
	SWE 7073	SWE 8854	SWE 15,927	
	FIN 2549	FIN 4387	FIN 6933	
% women	14.5%	16.0%	15.4%	*p* = 0.002
	SWE 15.4%	SWE 17.8%	SWE 16.7%	*p* < 0.001
	FIN 12.0%	FIN 12.4%	FIN 12.2%	*p* = 0.621
Mean age (all)	74.3 (SD 7.4)	70.8 (SD 7.5)	72.3 (SD 7.7)	*p* < 0.001
	SWE 73.8 (SD 7.3)	SWE 71.1 (SD 7.2)	SWE 72.3 (SD 7.4)	*p* < 0.001
	FIN 75.5 (SD 7.7)	FIN 70.3 (SD 8.1)	FIN 72.2 (SD 8.3)	*p* < 0.001
Mean age (men)	74.0 (SD 7.4)	70.5 (SD 7.5)	72.0 (SD 7.6)	*p* < 0.001
	SWE 73.6 (SD 7.3)	SWE 70.8 (SD 7.2)	SWE 72.1 (SD 7.4)	*p* < 0.001
	FIN 75.1 (SD 7.7)	FIN 69.8 (SD 7.9)	FIN 71.8 (SD 8.2)	*p* < 0.001
Mean age (women)	75.9 (SD 7.3)	72.7 (SD 7.2)	74.0 (SD 7.6)	*p* < 0.001
	SWE 75.0 (SD 7.2)	SWE 72.3 (SD 7.0)	SWE 73.4 (SD 7.2)	*p* < 0.001
	FIN 78.9 (SD 6.7)	FIN 73.8 (SD 8.5)	FIN 75.6 (SD 8.3)	*p* < 0.001
Ruptured AAA				
Number of operations	1470	7994	9464	
	SWE 1318	SWE 5949	SWE 7267	
	FIN 152	FIN 2045	FIN 2197	
% women	18.8%	16.4%	16.8%	*p* = 0.027
	SWE 18.9%	SWE 18.0%	SWE 18.2%	*p* = 0.449
	FIN 17.8%	FIN 11.8%	FIN 12.2%	*p* = 0.034
Mean age (all)	76.2 (SD 8.2)	73.6 (SD 8.0)	74.0 (SD 8.1)	*p* < 0.001
	SWE 76.3 (SD 8.1)	SWE 73.9 (SD 7.7)	SWE 74.3 (SD 7.9)	*p* < 0.001
	FIN 74.8 (SD 9.4)	FIN 72.7 (SD 8.7)	FIN 72.8 (SD 8.8)	*p* = 0.005
Mean age (men)	75.7 (SD 8.3)	73.0 (SD 8.0)	73.4 (SD 8.1)	*p* < 0.001
	SWE 75.9 (SD 8.1)	SWE 73.4 (SD 7.8)	SWE 73.9 (SD 7.9)	*p* < 0.001
	FIN 73.4 (SD 9.2)	FIN 72.0 (SD 8.6)	FIN 72.0 (SD 8.6)	*p* = 0.070
Mean age (women)	78.3 (SD 7.6)	76.4 (SD 7.4)	76.7 (SD 7.4)	*p* < 0.001
	SWE 77.9 (SD 7.6)	SWE 76.0 (SD 7.2)	SWE 76.4 (SD 7.3)	*p* < 0.001
	FIN 81.3 (SD 7.2)	FIN 78.0 (SD 7.7)	FIN 78.3 (SD 7.7)	*p* = 0.031

**Table 2:** Cox regression analysis of survival after the repair of an intact or ruptured AAA during 10-year follow-up. Hazard ratios and 95% confidence intervals are shown with corresponding *p*-values.

		HR	95% CI	*p* Value
AAA	Country (Finland vs. Sweden)	1.22	1.16–1.27	<0.001
	Female sex	0.93	0.88–0.99	0.020
	Age (10-year increments)	1.81	1.75–1.87	<0.001
	EVAR vs. OAR	1.21	1.15–1.27	<0.001
	Year of operation	0.97	0.97–0.98	<0.001
rAAA	Country (Finland vs. Sweden)	1.03	0.97–1.10	0.310
	Female sex	1.14	1.07–1.21	<0.001
	Age (10-year increments)	1.81	1.75–1.87	<0.001
	EVAR vs. OAR	0.81	0.75–0.87	<0.001
	Year of operation	0.98	0.98–0.99	<0.001

**Table 3:** Mean survival in years after intact or ruptured AAA repair according to age group. The bottom half of the table shows survival if the patient survived the first 90 days after the operation and 95% confidence intervals are shown.

	**Age**	**Mean Survival (Years), 95% CI**	
AAA	<65	13.22	12.90–13.53
	65–79	9.67	9.52–9.81
	≥80	6.84	6.57–7.11
RAAA	<65	10.29	9.82–10.77
	65–79	5.75	5.57–5.93
	≥80	2.59	2.44–2.74
If alive after first 90 days			
AAA	<65	13.49	13.17–13.81
	65–79	10.06	9.91–10.21
	≥80	7.28	7.00–7.57
RAAA	<65	13.07	12.59–13.55
	65–79	9.07	8.85–9.29
	≥80	5.53	5.30–5.79

## Data Availability

The data used in this study were not publicly available due to legislative, privacy and ethical restrictions. Data were accessed from national registries in Finland and Sweden, and their use was approved under specific research permits. Requests for data access can be directed to the corresponding author, Riku Pirinen, with appropriate ethical approval.

## Supplementary Materials

The following supporting information can be downloaded at https://www.mdpi.com/article/10.3390/jcm13154449/s1, Table S1: NCSP (Nomesco Classification of Surgical Procedures), 1998–2017.
