# Supplementary material for: The Outcome after Endovascular and Open Repair of Abdominal Aortic Aneurysms—A Binational Study Conducted between 1998 and 2017
Source: J Clin Med. 2024 Jul 29;13(15):4449. doi: 10.3390/jcm13154449 (PMC11313620; doi:10.3390/jcm13154449)
Supplement: Supplementary file 1 [file jcm-13-04449-s001.zip › jcm-3042249-supplementary.pdf]

Supplemental table s1.

NCSP *(Nomesco Classification of Surgical Procedures)*, 1998-2017

Open procedures

JAH 00 Laparotomy
PDA10 Exploration of infrarenal abdominal aorta
PCG10 Operation for aneurysm of supracoeliac or juxtarenal abdominal aorta
PDG10 Operation on infrarenal abdominal aorta for aneurysm
PDG20 Bypass from aorta to iliac artery for aneurysm
PDG21 Bypass from aorta to bilateral iliac arteries for aneurysm
PDG22 Bypass from aorta to iliac and contralateral femoral artery for aneurysm
PDG23 Bypass from aorta to femoral artery for aneurysm
PDG24 Bypass from aorta to bilateral femoral arteries for aneurysm
PDG99 Other operation for aneurysm of infrarenal abdominal aorta and iliac arteries
PDH10 Bypass from infrarenal abdominal aorta
PDH20 Bypass from aorta to iliac artery
PDH21 Bypass from aorta to bilateral iliac arteries
PDH22 Bypass from aorta to iliac and contralateral femoral artery
PDH23 Bypass from aorta to femoral artery
PDH24 Bypass from aorta to bilateral femoral arteries
PDH99 Other bypass from abdominal aorta or iliac artery
PDN10 Plastic repair of infrarenal abdominal aorta
PDW99 Other operation on infrarenal abdominal aorta and iliac arteries and distal connections

Endovascular procedures

PCQ10 Insertion of stent into suprarenal abdominal aorta
PDP10 Percutaneous plastic repair of infrarenal abdominal aorta
PDQ10 Insertion of stent into infrarenal abdominal aorta
PDQ21 Insertion of stent into infrarenal abdominal aorta and bilateral iliac arteries
PDS10 Endoscopic operation on infrarenal abdominal aorta
